# Conversion surgery for initially unresectable carcinoma of the ampulla of Vater following pathological complete response to chemotherapy: a case report

**DOI:** 10.1186/s40792-019-0680-z

**Published:** 2019-07-30

**Authors:** Yo Sato, Takanobu Hara, Yuko Takami, Yoshiyuki Wada, Tomoki Ryu, Shin Sasaki, Munehiro Yoshitomi, Seiya Momosaki, Masatoshi Murakami, Masayuki Hijioka, Toyoma Kaku, Ken Kawabe, Hideki Saitsu

**Affiliations:** 1 Department of Hepato-Biliary-Pancreatic Surgery, Clinical Research Institute, National Hospital Organization Kyushu Medical Center, 1-8-1 Jigyohama Chuo-ku, Fukuoka, 810-8563 Japan; 2 Department of Pathology, National Hospital Organization Kyushu Medical Center, Fukuoka, Japan; 3 Department of Gastroenterology, National Hospital Organization Kyushu Medical Center, Fukuoka, Japan

**Keywords:** Carcinoma of the ampulla of Vater, Chemotherapy, Conversion surgery

## Abstract

**Background:**

Carcinoma of the ampulla of Vater with distant metastases is regarded as unresectable. Systemic chemotherapy is basically the treatment of choice for such tumors.

**Case presentation:**

A 68-year-old woman was referred to our hospital and diagnosed with carcinoma of the ampulla of Vater with lymph node and multiple liver metastases. She underwent systemic chemotherapy with a combination of gemcitabine and cisplatin. After 19 months of treatment, the primary tumor and liver metastases were difficult to detect on follow-up images. Shrinkage of the enlarged lymph nodes was also confirmed. Surgical resection was performed with curative intent after a multidisciplinary meeting. Pathological examination of the resected specimen showed no residual tumors. Systemic chemotherapy achieved a pathological complete response. The postoperative course was uneventful, and the patient remained free of recurrent disease at 10 months of follow-up.

**Conclusion:**

This case shows the possibility of conversion surgery after systemic chemotherapy for carcinoma of the ampulla of Vater.

## Background

Complete surgical resection is the only potential curative treatment for carcinoma of the ampulla of Vater (AC) [1]. Previous reports have indicated that the possibility of curative resection was 50% [2] and that the 5-year survival rate ranged from 33 to 66% [3–7]. However, tumors with distant metastasis such as liver metastasis, para-aortic lymph node metastasis, or peritoneal carcinomatosis are regarded as unresectable. Palliative chemotherapy is often administered to these patients. We herein report a case of AC with synchronous liver metastasis in which a pathological complete response (CR) was obtained by chemotherapy.

## Case presentation

**Fig. 1:**
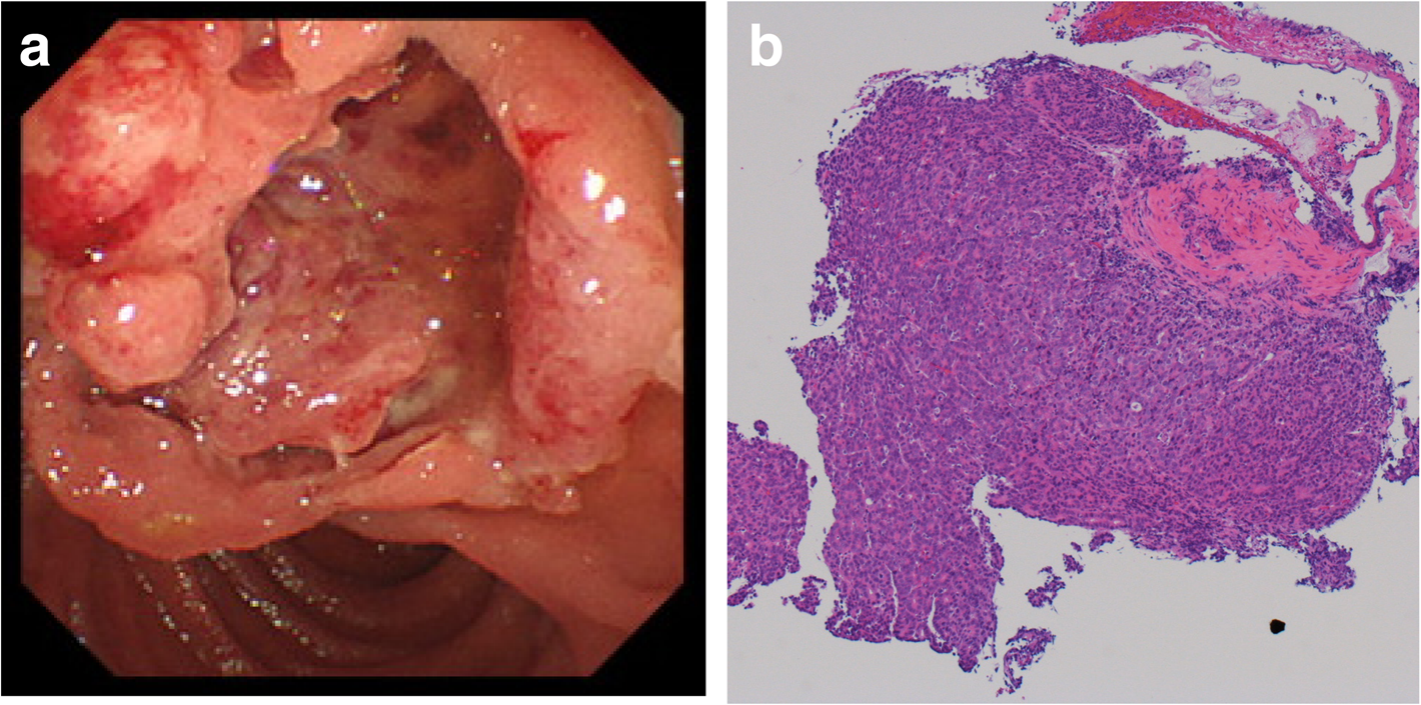
**a** Endoscopy revealed an irregularly shaped ulcerated tumor in the ampulla of Vater. **b** The biopsy specimen suggested moderately to poorly differentiated adenocarcinoma

**Fig. 2:**
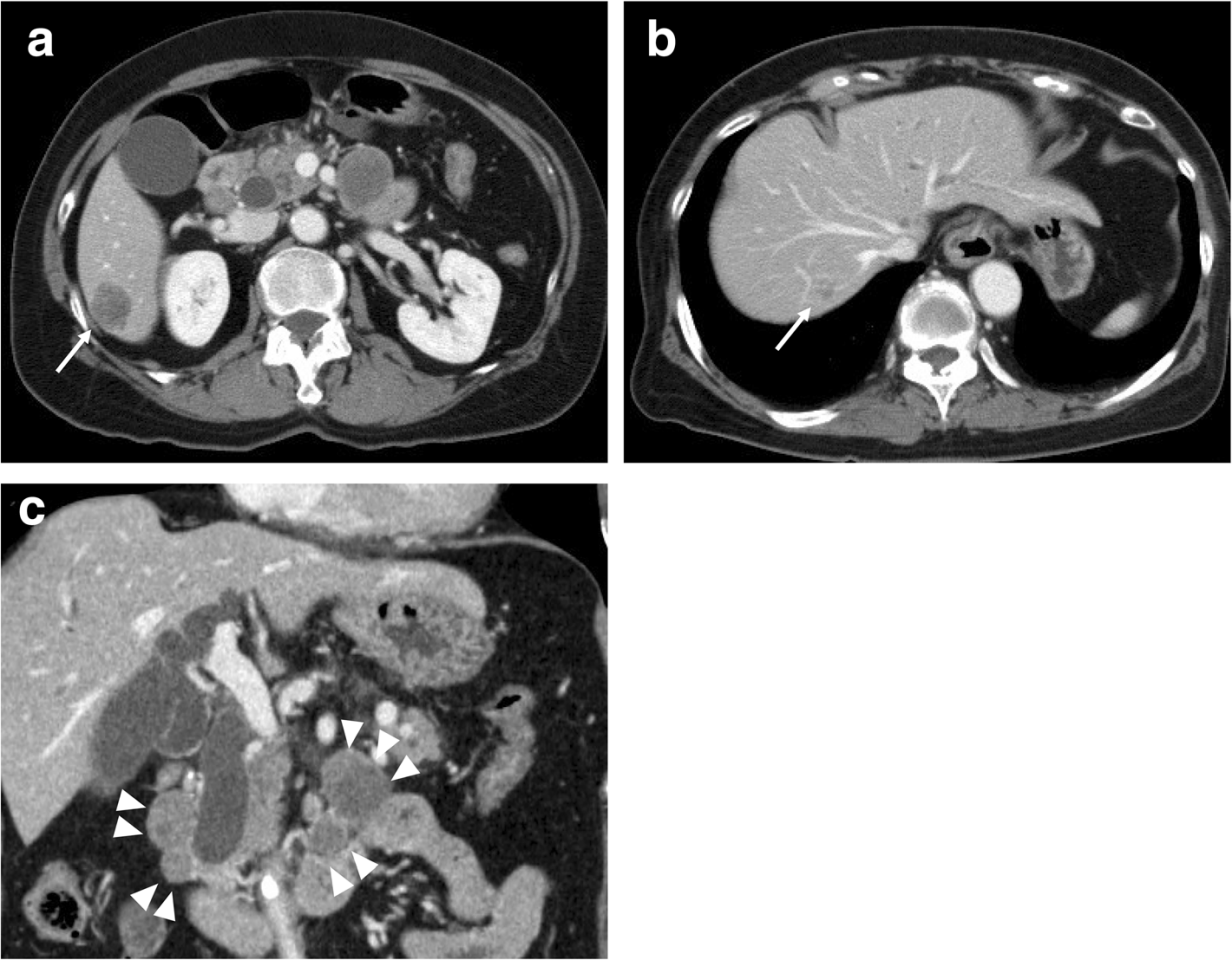
**a**, **b** Abdominal computed tomography showed dilatation of the intrahepatic and extrahepatic bile duct. Two hypovascular lesions were detected in the liver (arrow). **c** Multiple enlarged lymph nodes were present around the distal bile duct and superior mesenteric artery (arrowhead)

A 68-year-old woman was admitted to the hospital because of melena and epigastric pain. She had a medical history of hypertension, dyslipidemia, and psoriasis vulgaris for which she was undergoing treatment with prednisolone (1 mg/day). The patient’s serum amylase and lipase levels were elevated at 2238 IU/L and 3900 U/L, respectively. Her white blood cell count was 9700/μL, and her C-reactive protein level was 0.61 mg/dL. Tumor markers such as carcinoembryonic antigen and carbohydrate antigen 19-9 were within normal limits. Upper gastrointestinal endoscopy showed an irregular ulcerative tumor located at the ampulla of Vater. Pathological examination of the tumor showed severely atypical epithelial cells arranged in sheets and vague glands. The feature suggested moderately to poorly differentiated adenocarcinoma (Fig. 1a, b). Computed tomography (CT) revealed a hypovascular tumor of 32 mm in size at the head of the pancreas. The common bile duct and pancreatic duct were dilated. In addition, two hypovascular masses were found in the liver: a 1-cm mass at segment 7 and a 2-cm mass at segment 6. The regional lymph nodes along the superior mesenteric artery (SMA) (#14d) and the right side of the common bile duct (#12b2) had swollen to 3 cm and 2 cm in size, respectively (Fig. 2a–c).

**Fig. 3:**
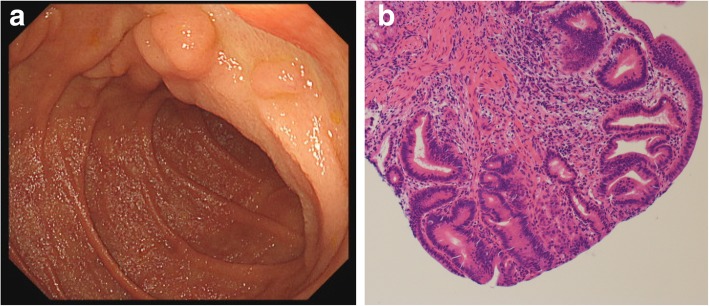
**a** The ulcerative tumor could not be detected by follow-up endoscopy after 19 months of chemotherapy. **b** A biopsy specimen showed papillotubular structures with fibrous stroma. A few small, irregularly shaped ductal structures were present without invasion that suggested high-grade adenoma

**Fig. 4:**
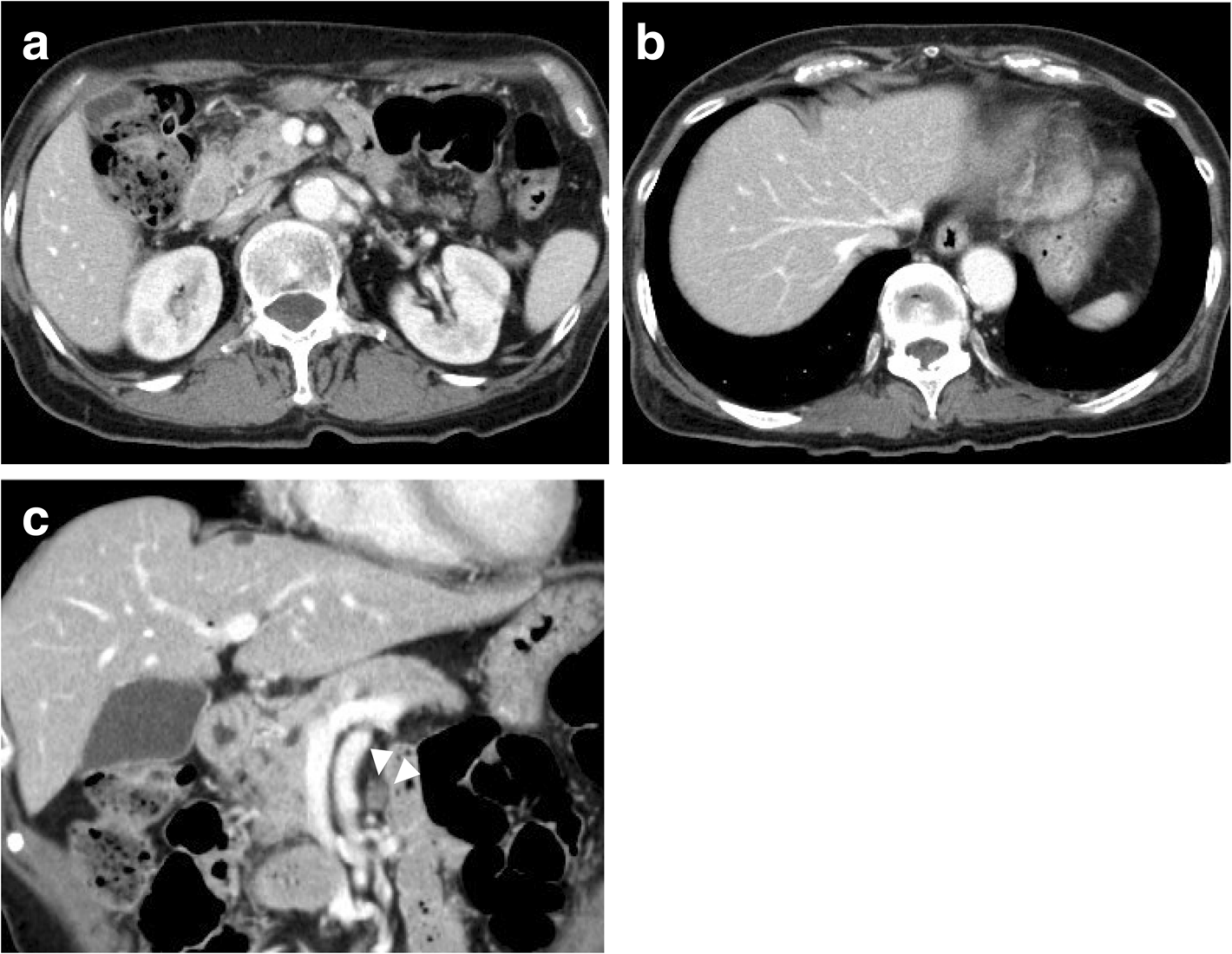
**a**, **b** Abdominal computed tomography after 19 months of chemotherapy showed disappearance of multiple liver tumors. **c** Chemotherapy also contributed to the reduction in the size of the enlarged lymph nodes around the bile duct and superior mesenteric artery (arrowhead)

Given these findings, the patient was diagnosed with AC with lymph node and synchronous liver metastases. Because the disease was so advanced, surgical treatment was not feasible. First, she was treated for acute pancreatitis by stent placement into bile duct and pancreatic duct. Chemotherapy with gemcitabine (GEM) and cisplatin (CDDP) was then performed at dosages of 1000 mg/m^2^ and 25 mg/m^2^ weekly for 2 weeks, followed by a 1-week rest. Four months later, grade 2 bone marrow suppression occurred and was successfully managed by reducing the administered doses to 80%. After 6 months, the chemotherapy frequency was changed to biweekly administration. Because follow-up CT revealed shrinkage of the liver metastases and enlarged lymph nodes, we decided to continue the treatment. The liver metastases became difficult to identify at the 14-month CT scan. Because of grade 3 thrombocytopenia, the CDDP was reduced to 60% of the original dose and then stopped. GEM was reduced to 50% of the original dose and continued as monotherapy. Upper gastrointestinal endoscopy at 19 months from treatment initiation showed only a 0-IIa–like bulge in the papillary area, and the biopsy result was high-grade adenoma (Fig. 3a, b). Abdominal CT showed significant shrinkage of the main tumor. The liver metastases had almost vanished and were difficult to identify. The enlarged lymph nodes (#14d) had shrunk but remained present (Fig. 4a–c). However, fluorodeoxyglucose positron emission tomography/CT showed no accumulation in the primary tumor, liver metastases, or lymph nodes.

**Fig. 5:**
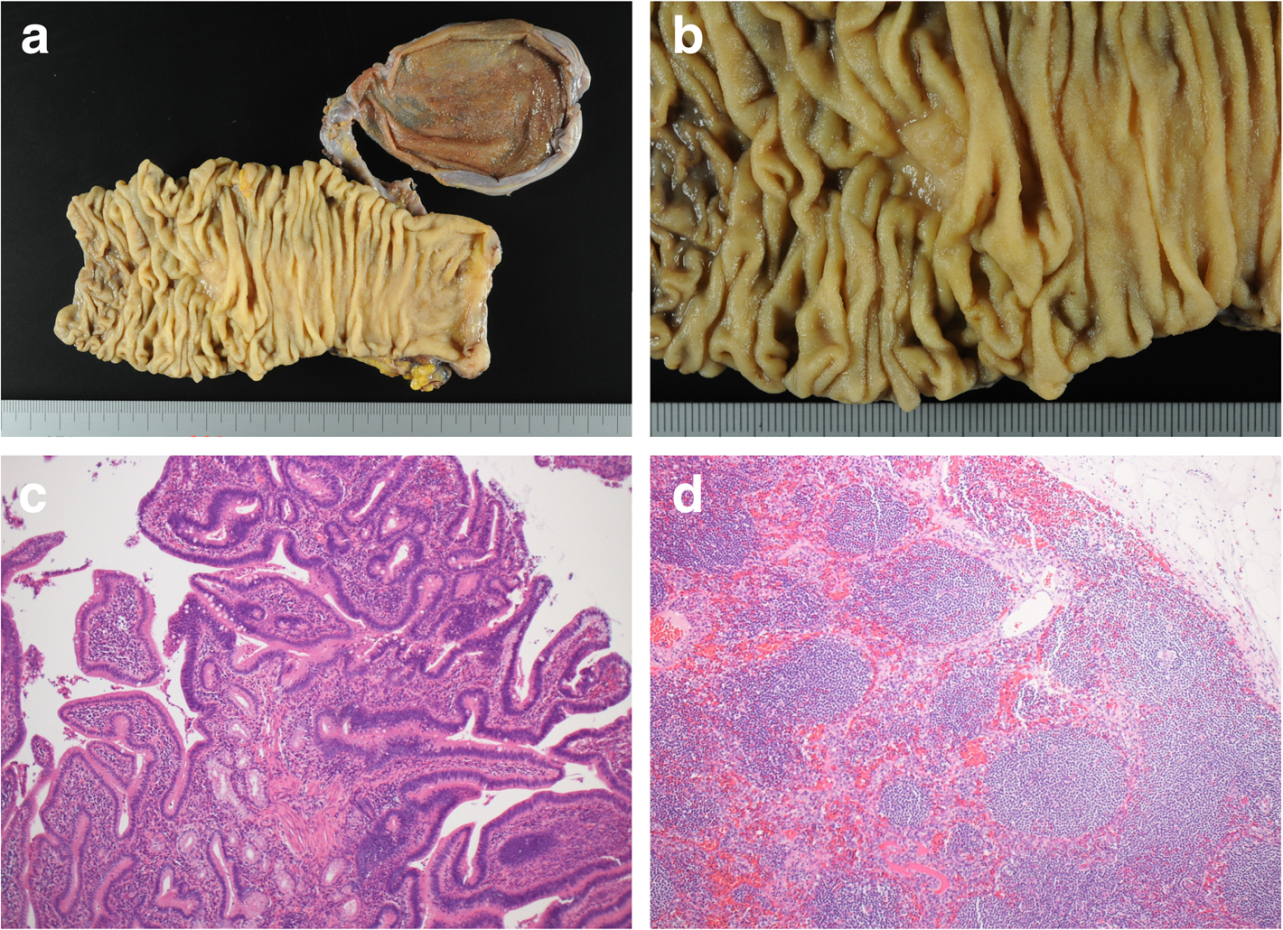
**a**, **b** Macroscopic findings of the resected specimen. **c** Histopathological examination demonstrated ampullary mucosa with moderately atypical glands. The polarity of the nuclei was basically conserved. **d** Resected lymph nodes around the superior mesenteric artery consisted of hyperplasia of lymphoid follicles and necrotic tissue. There were no signs of residual tumor cells

The effect of chemotherapy almost achieved CR according to the radiological and pathological findings. After a multidisciplinary meeting, the patient was considered a surgical candidate. Twenty-one months after the initial diagnosis and induction of chemotherapy, she underwent subtotal stomach-preserving pancreaticoduodenectomy with D2 lymph node dissection. Lymph node along the SMA (#14d) detected preoperatively were harvested with preserving the SMA nerve plexus. This was the choice to reduce the possibilities of serious postoperative complications. No metastatic liver tumors were detected by intraoperative sonography; therefore, liver resection was not performed. Pathological examination of the resected specimen demonstrated no residual carcinoma in the ampulla of Vater, distal bile duct, or pancreatic head. The definitive diagnosis was intestinal-type adenoma. An enlarged lymph node located around the SMA was composed of necrotic tissue and showed no signs of residual tumor cells (Fig. 5a–d).

The postoperative course was uneventful, and the patient was discharged 20 days after surgery. Adjuvant chemotherapy was not performed because the patient had already received long-term preoperative chemotherapy and had no signs of residual tumors. The patient remained free of recurrent disease at the 10-month follow-up.

## Discussion

AC is relatively rare, and the reported incidence is around 7% of all periampullary tumors [6]. The frequency of adenocarcinoma is the highest among all malignant tumors, and differentiation from bile duct cancer, pancreatic cancer, and duodenal cancer infiltration is required. According to the Japanese registry of biliary tract cancer, the resectability rate of AC is 89.4% and the curative resection rate is 93.0%. The 5-year survival rate after resection is 52.8%, which is higher than that of cholangiocarcinoma (32.7%) or gallbladder cancer (41.6%). Thus, AC has a relatively good prognosis. However, patients with distant metastasis have a poor prognosis, and the 5-year survival rate is 0.0% [5]. Chemotherapy is the first choice for advanced AC with distant metastases. According to a recent randomized phase III study from the UK (ABC-02 trial), the combination of GEM and CDDP (GC) has become the current standard for unresectable biliary tract cancer [8]. A Japanese multicenter phase III trial also demonstrated the advantage of GC over GEM alone [9]. In the present case, we started the GC regimen with palliative intent, not as curative treatment. However, a dramatic effect was observed for both the primary and metastatic tumors. Disappearance of the liver metastasis and benign results of the biopsy from the ampulla of Vater offered hope of curative resection.

We searched previous reports and found four case reports describing curative resection of initially unresectable AC due to distant metastases [10–13]. All reports described favorable outcomes. However, to the best of our knowledge, this is the first report of successful conversion surgery for AC following the achievement of pathological CR in the primary tumor and metastatic lesions.

Recent advances in chemotherapy have led to several cases of successful conversion surgery in patients with advanced biliary tract cancer or pancreatic cancer, as for patients with colorectal cancer [14–16]. However, because no comprehensive reports are available, no consensus has been reached regarding the timing of resection, its impact on the prognosis, or the need for postoperative adjuvant therapy. Because of the rarity of the disease, performing well-powered clinical trials of conversion surgery after chemotherapy in patients with AC will be much more limited. We consider that surgical options should be discussed among a multidisciplinary team, at least when dramatic effects of chemotherapy are confirmed, as in our case. Further collection of additional cases is expected.

Because the liver metastasis could not be confirmed during surgery in the present case, there was a concern that pathological CR could not be demonstrated for the liver metastasis. Postoperative adjuvant therapy was not performed because of the long-term neoadjuvant chemotherapy and pathological CR of the primary tumor and metastatic lymph nodes. We will continue close follow-up with particular attention to relapse of liver metastasis.

## Conclusions

We have herein reported a case of conversion surgery after chemotherapy for an initially unresectable AC with synchronous multiple liver metastases. The possibility of conversion surgery may increase with the development of chemotherapies. Collection of additional successful cases and further investigation are required to evaluate the safety, feasibility, and advantages of this approach.

## Data Availability

Data will be made available from the corresponding author upon request.

## Abbreviations

AC: Carcinoma of the ampulla of Vater
CDDP: Cisplatin
CR: Complete response
CT: Computed tomography
GC: Gemcitabine and cisplatin
GEM: Gemcitabine
SMA: Superior mesenteric artery

## References

[1] Miyazaki M, Ohtsuka M, Miyakawa S, Nagino M, Yamamoto M, Kokudo N (2015). Classification of biliary tract cancers established by the Japanese Society of Hepato-Biliary-Pancreatic Surgery: 3(rd) English edition. J Hepatobiliary Pancreat Sci.

[2] Romiti A, Barucca V, Zullo A, Sarcina I, Di Rocco R, D'Antonio C (2012). Tumors of ampulla of Vater: a case series and review of chemotherapy options. World J Gastrointest Oncol.

[3] Balachandran P, Sikora SS, Kapoor S, Krishnani N, Kumar A, Saxena R (2006). Long-term survival and recurrence patterns in ampullary cancer. Pancreas..

[4] de Castro SM, van Heek NT, Kuhlmann KF, Busch OR, Offerhaus GJ, van Gulik TM (2004). Surgical management of neoplasms of the ampulla of Vater: local resection or pancreatoduodenectomy and prognostic factors for survival. Surgery..

[5] Miyakawa S, Ishihara S, Horiguchi A, Takada T, Miyazaki M, Nagakawa T (2009). Biliary tract cancer treatment: 5,584 results from the Biliary Tract Cancer Statistics Registry from 1998 to 2004 in Japan. J Hepato-Biliary-Pancreat Surg.

[6] O'Connell JB, Maggard MA, Manunga J, Tomlinson JS, Reber HA, Ko CY (2008). Survival after resection of ampullary carcinoma: a national population-based study. Ann Surg Oncol.

[7] Riall TS, Cameron JL, Lillemoe KD, Winter JM, Campbell KA, Hruban RH (2006). Resected periampullary adenocarcinoma: 5-year survivors and their 6- to 10-year follow-up. Surgery..

[8] Valle J, Wasan H, Palmer DH, Cunningham D, Anthoney A, Maraveyas A (2010). Cisplatin plus gemcitabine versus gemcitabine for biliary tract cancer. N Engl J Med.

[9] Okusaka T, Nakachi K, Fukutomi A, Mizuno N, Ohkawa S, Funakoshi A (2010). Gemcitabine alone or in combination with cisplatin in patients with biliary tract cancer: a comparative multicentre study in Japan. Br J Cancer.

[10] Ohno T, Koguchi H, Miura A, Tanaka Y, Endo M, Matsunaga S (2009). A case of advanced ampullary carcinoma successfully resected after primary chemotherapy with s-1 and gemcitabine. Gan To Kagaku Ryoho.

[11] Shimada T, Sakata J, Yamamoto J, Usui K, Naito T, Tani T (2015). Surgical resection after chemotherapy for ampullary carcinoma with synchronous liver metastasis--report of a case. Gan To Kagaku Ryoho..

[12] Fujii W, Hayashi K, Yamada S, Kusanagi H (2016). Curative resection of carcinoma of the ampulla of Vater with lymph node metastases around the abdominal aorta after chemotherapy: a case report. Int J Surg Case Rep.

[13] Tanaka R, Yamada N, Kinoshita H, Nishimura S, Taenaka N (2017). A case of resected ampulla of Vater cancer with a hepatic metastasis after neoadjuvant chemotherapy. J Jpn Surg Assoc.

[14] Watanabe T, Furuse J, Okano N, Suzuki Y, Kamma H, Sugiyama M (2017). A pathological complete response after combined chemotherapy of gemcitabine and S-1 in advanced biliary tract cancer with para-aortic lymph nodes metastasis: a case report. Surg Case Rep.

[15] Furukawa K, Uwagawa T, Sakamoto T, Shiba H, Tsutsumi J, Yanaga K (2015). Curative resection after gemcitabine, cisplatin and S-1 chemotherapy for initially unresectable biliary duct cancer: a case report. Anticancer Res.

[16] Furuse J, Shibahara J, Sugiyama M (2018). Development of chemotherapy and significance of conversion surgery after chemotherapy in unresectable pancreatic cancer. J Hepatobiliary Pancreat Sci..

